# Editorial: Supramolecular Assembly Based Functional Nanostructures for Biomedical Applications

**DOI:** 10.3389/fchem.2020.637926

**Published:** 2021-01-28

**Authors:** Tianjiao Ji, Kai Han, Huaimin Wang

**Affiliations:** ^1^ CAS Key Laboratory for Biomedical Effects of Nanomaterials and Nanosafety, CAS Center for Excellence in Nanoscience, National Center for Nanoscience and Technology, Beijing, China; ^2^ Center of Materials Science and Optoelectronics Engineering, University of Chinese Academy of Sciences, Beijing, China; ^3^ Department of Pharmaceutical Sciences, University of Michigan, Ann Arbor, MI, United States; ^4^ Key Laboratory of Precise Synthesis of Functional Molecules of Zhejiang Province, School of Science, Westlake University, Westlake Institute for Advanced Study, Zhejiang, China

**Keywords:** supramolecular assembly, nanostructures, biomedical applications, imaging, therapy

**Editorial on the Research Topic**

**Supramolecular Assembly Based Functional Nanostructures for Biomedical Applications**

Supramolecular chemistry is an important branch of chemistry. Rationally designed molecules could spontaneously assemble to uniform nanostructures through supramolecular interactions (hydrogen bond, hydrophobic forces, Van der Waals forces, electrostatic interaction, *etc*.). For example, fatty acid–modified dexamethasone (Dex) could self-assemble to nanostructures (nanofibers or nanoparticles) through intermolecular interactions (probably through hydrophobic forces, hydrogen bond, and Van der Waals forces), and different (fatty acids) modification significantly influenced the morphologies and gelation behaviors (Liu et al.). Types of supramolecular interactions were described in the review article of Chen and his coworkers in this topic.

Taking advantages of supramolecular-based assembly, many pharmaceutical issues, such as poor solubility and short half-life of drugs under physiological conditions, can be solved. For example, poor water-soluble properties of rapamycin (RAPA, a promising drug for alleviating hepatic steatosis) limited its clinical use; however, its dispersion and bioavailability were significantly improved when encapsulated in mPEG-PLGA (through hydrophobic forces) (Zhao et al.); baicalein (BCL) is also a poor-solubility drug, but Zhang et al. prepared BCL nanocrystals (co-assembling a cationic beta-lactoglobulin with BCL) that exhibited long-term stability in physiological conditions; the half-life of nucleic acid drugs can be prolonged if they are encapsulated/absorbed in nanocarriers, which are usually obtained through electrostatic interactions, as reported by Xu et al. in this topic.

The biomedical functions of supramolecular assemblies can be determined either by the carrier molecules or by the encapsulated drugs. Zhang et al. and Jin et al. reviewed types of functional peptide-based supramolecular assemblies and their applications in cancer therapy. Li group developed an amphiphilic molecule by conjugating photosensitizer protoporphyrin IX (PpIX) and the FAM/Dabcyl-based Förster resonance energy transfer (FRET) donor–acceptor through a PEG linker, and this molecule self-assembled to nanoparticles that were able to image hypoxia environment of tumors and to achieve photodynamic therapy.

Several works (in this topic) are related to immunotherapy because immunotherapy represents a new revolution in cancer therapy and becomes a superhot topic currently. Supramolecular assembly nanostructures exhibit great potential in terms of therapeutic vaccine, immune microenvironment modulation, *etc*. The review from Chen et al. underlined the advantages of tunable, modular, and reversible supramolecular assemblies in the loading/release of adjuvant, antigen, proteins, and drugs. It highlighted different supramolecular assemblies for cancer immunotherapy including supramolecular peptide assembly, DNA assembly, lipid hydrophobic assembly, host–guest assembly, as well as biomolecular recognition assembly. Supramolecular hydrogels can deliver antigen to modulate immunity, and Liu et al. developed reduction-responsive co-assembled hydrogels with different surface properties *In vivo* studies demonstrated that positively charged K-vac boosted significantly higher antibody production and antitumor immune responses. Supramolecular assembly can also modulate the T-cell function, and Xie et al. described that supramolecular photothermal ICG-liposome nanoparticles with light irradiation can increase the expression of immune checkpoint biomarkers, that is, anti-program death-1 (anti-PD1) and anti–T-cell immunoglobulin and mucin domain-containing protein 3 (anti-TIM-3) monoclonal antibodies, in tumor-infiltrating CD8 T cells. Consequently, the combination with anti–PD-1 and anti–TIM-3 can inhibit both primary and distant tumor growth.

In summary, this topic reports functional nanostructures (liposome, micelle, nanofibers, *etc*.) by making full use of supramolecular self-assembly techniques. The prepared nanostructures can overcome some drawbacks (e.g., lack of targeting, low encapsulation efficiency, complex preparation procedures, *etc*.) of current nanomaterials, which can provide many feasible references for designing translational biomaterials.

## Author Contributions

TJ, KH, and HW discussed and wrote the manuscript.

## Conflict of Interest

The authors declare that the research was conducted in the absence of any commercial or financial relationships that could be construed as a potential conflict of interest.

